# Multifunctional aloe vera-based biomaterial for vital pulp therapy: an in vivo evaluation

**DOI:** 10.1007/s10266-025-01258-3

**Published:** 2025-11-17

**Authors:** Sharon S. Namazi, Pedro Henrique Chaves de Oliveira, Neville J. McDonald, Bruno Cavalcanti, Hajime Sasaki, Marco C. Bottino, Renan Dal-Fabbro

**Affiliations:** 1 Department of Cariology, Restorative Sciences, and Endodontics, School of Dentistry, University of Michigan, 1011 N. University Ave, Ann Arbor, MI 48109 USA; 2 Department of Preventive and Restorative Dentistry, School of Dentistry, São Paulo State University, Araçatuba, São Paulo Brazil; 3 Department of Biomedical Engineering, College of Engineering, University of Michigan, Ann Arbor, MI USA

**Keywords:** Aloe vera, Electrospinning, Gelatin methacryloyl, Regenerative endodontics

## Abstract

This study aimed to evaluate the effects of Gelatin methacryloyl/Aloe vera (GelMA/AV) scaffolds, both alone and in combination with mineral trioxide aggregate (MTA), on pulp inflammatory response and mineralized tissue formation in a rat model of pulp exposure. GelMA/AV nanofibers (70:30) were fabricated via electrospinning and photocrosslinked. Twenty-four rats were randomly assigned to three groups (*n* = 8/ group): GelMA/AV, GelMA/AV + MTA, and MTA (control). Following pulp exposure of the first upper molars (*n* = 2/animal), the materials were placed over the exposed pulp as the pulp capping agent, and MTA was applied or not, depending on the group. The cavities were then sealed with glass ionomer. Animals were euthanized at 7 or 28 days post-procedure for histological, immunohistochemical, and micro-computed tomography (micro-CT) analyses. Data were analyzed using one-way or two-way ANOVA at a 5% significance level. Micro-CT analysis at 28 days revealed new hard tissue formation in the pulp chamber across all groups. GelMA/AV + MTA demonstrated comparable mineralized volume and tissue mineral density to MTA. Histologically, the GelMA/AV + MTA group exhibited a significantly lower cell count than the MTA and GelMA/AV groups at 28 days. However, for macrophage polarization, the GelMA/AV + MTA exhibited a similar expression of CD163-positive (M2) and iNOS-positive (M1) cells compared to the MTA. Lastly, GelMA/AV + MTA also showed expression of nestin and DMP1 at 28 days, indicating active mineralization. In conclusion, a thin GelMA/AV sublayer beneath MTA preserves MTA-driven mineralization but does not reduce pulp inflammation or enhance mineralization beyond MTA in this model. Optimization of AV dosing/formulation and longer follow-up are necessary before clinical translation.

## Introduction

Direct pulp capping (DPC), a key vital pulp therapy (VPT) approach, involves placing a biocompatible, bioactive material over the exposed pulp to stimulate tertiary (reparative) dentin formation, thus creating a dentin bridge that maintains tooth vitality [1, 2]. Calcium silicate cements (CSCs), such as mineral trioxide aggregate (MTA) and Biodentine (BD), are considered the gold standard for DPC due to their excellent sealing ability, biocompatibility, and capacity to induce calcific bridge formation [3]. However, despite high clinical success rates, CSCs still have inherent limitations at the tissue level. For example, while these materials often induce a dentin bridge, they lack antimicrobial efficacy and have only limited anti-inflammatory (immunomodulatory) potential. In other words, they do not actively resolve persistent pulp inflammation or eradicate residual bacteria, which may help explain why regenerative outcomes are not uniformly ideal in every case [4–6].

The prolonged release of inflammatory mediators can disrupt the stem cell microenvironment, hindering regenerative processes [7, 8]. To overcome these challenges, regenerative endodontics has increasingly explored novel local biomaterials with anti-inflammatory properties to create a conducive environment that favors regenerative responses. In parallel, recent research has focused on natural compounds and plant-based substances to address the shortcomings of commonly used synthetic drugs, such as inactivity over time, drug resistance, potential cytotoxicity, and adverse effects on dentin [9].

Aloe vera (AV), derived from *Aloe barbadensis Miller*, has emerged as a promising natural compound for tissue engineering applications due to its broad-spectrum antibacterial, anti-inflammatory, immunomodulatory, and low-toxicity properties [10–12]. AV has been shown to exert its anti-inflammatory activity by suppressing pro-inflammatory cytokines, including TNF-α, IL-6, IL-1β, and MMP-9, as well as reducing neutrophil migration, edema formation, and tissue destruction. The anti-inflammatory effects of AV on lipopolysaccharide (LPS)-activated human macrophages have also been investigated, demonstrating significant downregulation of IL-1β via suppression of the NF-κB signaling pathway [13].

Gelatin methacryloyl (GelMA) has been widely used in fabricating scaffolds for pulp regeneration due to its cytocompatibility, hydrophilicity, and cell adhesion properties [14]. Building on these advantages, a photocrosslinkable GelMA nanofiber scaffold incorporating AV (GelMA/AV) has recently been developed as a stable platform for the controlled delivery of AV’s bioactive components. In vitro, GelMA/AV (70:30) nanofibers exhibited significant antimicrobial activity against *E. faecalis*, sustained biofilm reduction, minimal cytotoxicity, and immunomodulatory effects favoring anti-inflammatory macrophage (M2) polarization, suggesting potential for managing chronic inflammation in pulpitis [9]. Here, these findings were examined in vivo using GelMA/AV alone and in combination with mineral trioxide aggregate (MTA) in a well-established rat pulp capping model. The central hypothesis is that a thin GelMA/AV scaffold placed beneath MTA will deliver localized anti-inflammatory/immunomodulatory cues, while MTA provides a mineralizing stimulus and coronal seal, together, fostering a pro-healing immune milieu and promoting a more continuous dentin bridge, improving clinically relevant outcomes.

## Materials and methods

### GelMA/AV scaffold preparation

GelMA was synthesized using Type A gelatin from porcine skin (SLBM9945V, Sigma-Aldrich, St. Louis, MO, USA) and methacrylic anhydride (276,685, Sigma-Aldrich) as previously described [9]. Commercially available aloe vera gel powder (Minature™, Batch #00823Marudhar Impex, Ahmedabad, India) was incorporated into GelMA at a 70:30 ratio (GelMA/AV). Nanofibers were fabricated via electrospinning, followed by photocrosslinking to ensure stability. The resulting GelMA/AV (70:30) scaffolds were UV sterilized (30 min on each side) and stored under sterile conditions until use.

### Animal model and experimental design

All animal procedures were conducted in accordance with Institutional Animal Care and Use Committee guidelines (PRO00010911). The sample size was calculated based on previous studies [15]. Twenty-four rats were randomly allocated to three experimental conditions (*n* = 8 per group): (1) GelMA/AV nanofiber scaffold, (2) GelMA/AV scaffold overlaid with mineral trioxide aggregate (GelMA/AV + MTA), and (3) MTA alone (control). Under general anesthesia, induced by an intraperitoneal injection of ketamine (50 mg/kg; Hospira, Lake Forest, IL, USA) and xylazine (5 mg/kg; Akorn, Lake Forest, IL, USA), standardized pulp exposures were prepared in the first maxillary molars (occlusal surface) using a No. ½ dental round burr (Kavo Kerr Group, USA), ensuring the exposure matched the bur’s full diameter. The assigned capping material was applied immediately using an endodontic explorer and compacted with sterile paper points (Absorbent paper points, DENTSPLY Maillefer, Tulsa, OK, USA): in group 1, GelMA/AV nanofibers were directly applied to the exposure site; in group 2, the scaffold was covered with a thin MTA layer (MTA Angelus White, Londrina, PR, Brazil); and in group 3, only MTA was used. Each cavity was then sealed with a glass ionomer restorative (Ketac Molar; 3 M, Seefeld, Germany). Animals were euthanized by CO_2_ overdose at either 7 or 28 days post-treatment.

### Micro-CT analysis

At the 28 day time point, the maxillary segments were harvested, fixed in 10% neutral-buffered formalin for 24 h, and then transferred to 70% ethanol. Specimens were scanned using a high-resolution micro-CT system (Scanco CT 100 system, Scanco Medical AG, 70 kV, 114 µA, 8 µm voxel size). An experienced examiner quantified the hard tissue formation (mm^3^) and tissue mineral density (TMD, g/cm^3^) within the newly formed hard tissue occupying the pulp chamber. The region of interest (ROI) was defined as the pulp chamber volume extending from immediately beneath the capping material apically to the cervical third (the level at which the canal orifices begin), distant from the chamber and root dentin walls, to exclude pre-existing dentin. Within this ROI, only voxels falling within a dentin-equivalent density window were quantified as dentin-like tissue. This segmentation isolates mineralization arising from the capped exposure rather than native dentin surfaces and reflects a widely adopted micro-CT approach. A global threshold of 320–600 grayscale units was applied uniformly to all samples, thereby excluding MTA’s intrinsic radiopacity and allowing quantification of only the neoformed dentin [15, 16].

### Histological and immunohistochemical analysis

Specimens harvested at 7 and 28 days were immersed in 10% neutral-buffered formalin for 24 h, then decalcified in 10% EDTA (pH 7.4) for 6 weeks. After routine dehydration and paraffin embedding, 5 µm sections were prepared. Hematoxylin–eosin staining was used to evaluate inflammatory infiltrates. At 100 × magnification (Echo Revolve, Discover Echo Inc., San Diego, CA, USA), inflammatory cells were quantified in ImageJ by applying a binary threshold (10–130), circularity filter (0.3–1.0), and a size range of 10–30 µm. Cell counts were normalized to pulp chamber area (cells/mm^2^) obtained from the same images. Immunofluorescence was performed on 28 day sections to assess mineralization and macrophage phenotype. Slides were incubated overnight at 4 °C with the following primary antibodies (1:100): anti-DMP1 (PA5-47,621, Invitrogen) and anti-Nestin (19,483–1-AP, Proteintech) for mineralization, and anti-iNOS (ab283655, Abcam) and anti-CD163 (ab182422, Abcam) for M1 and M2 macrophage markers, respectively. The corresponding secondary antibodies were applied for 1 h at room temperature: Goat Anti-Mouse IgG Alexa Fluor 488 for Nestin and CD163, and Goat Anti-Mouse IgG Alexa Fluor 594 for iNOS and DMP1, followed by VECTASHIELD with DAPI (Vector Laboratories) for nuclear counterstaining. Fluorescent images captured at 100 × were processed in ImageJ by channel separation, threshold adjustment, and area normalization to calculate the density of marker-positive cells [15, 17, 18].

### Statistical analysis

Data are reported as mean ± standard deviation. Normality was assessed for each dataset; variables that satisfied normality assumptions were analyzed with one-way or two-way ANOVA, followed by Tukey’s post hoc test. When normality was not met, the Kruskal–Wallis test with Dunn’s post hoc correction was applied. All analyses were performed in GraphPad Prism v10.4.2 (GraphPad Software, San Diego, CA). Statistical significance was set at *p* < 0.05.

## Results

### Micro-CT analysis of hard tissue formation

**Fig. 1:**
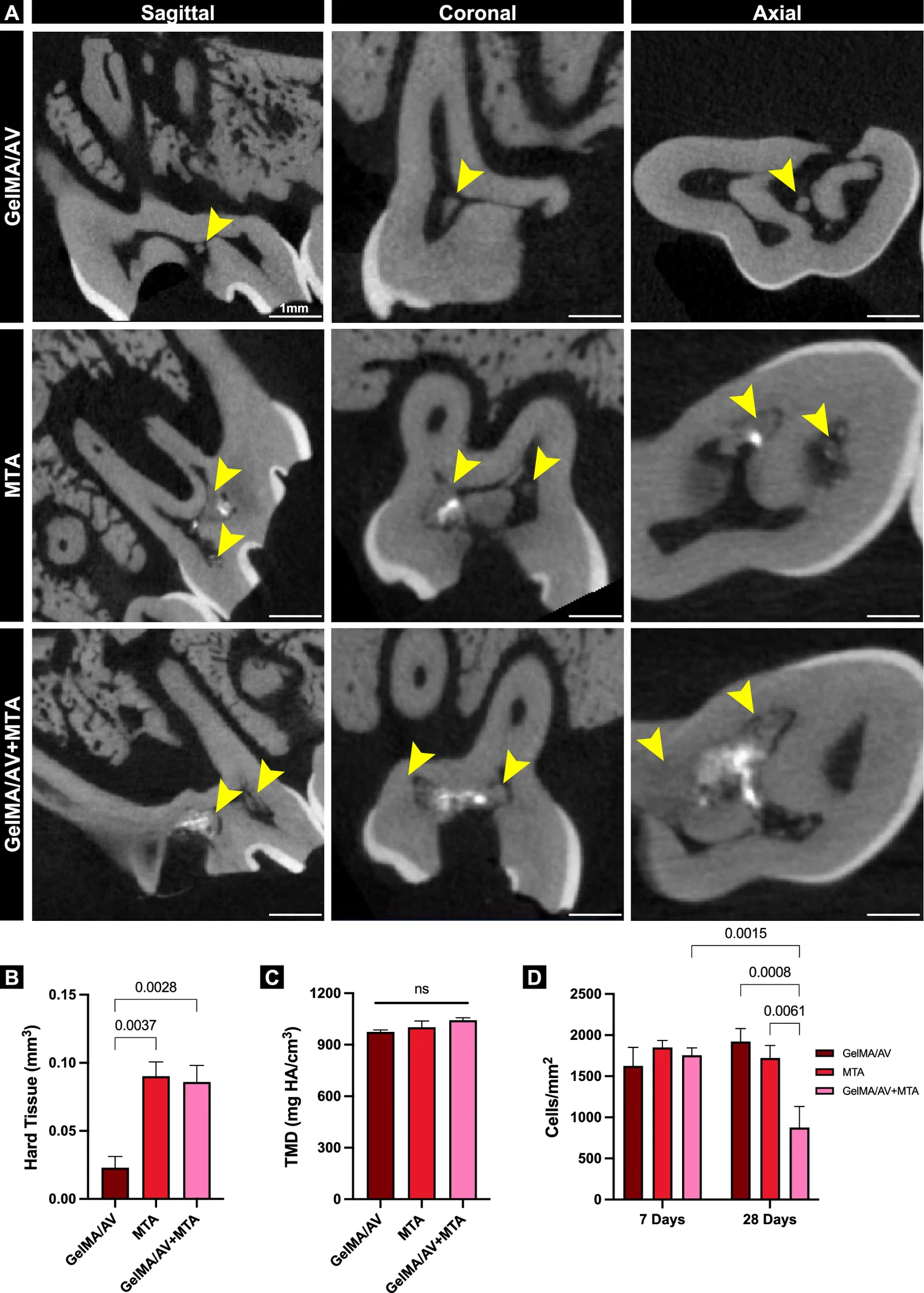
Micro-CT evaluation of hard tissue formation 28 days after pulp capping. **A** 3-D reconstructions of the pulp chamber from three orthogonal views; newly deposited dentin-like tissue is highlighted by yellow arrowheads. Scale bar: 1 mm. **B** Hard tissue in mm^3^, and **C** tissue mineral density (TMD, g/cm^3^) were compared across experimental groups using one-way ANOVA followed by Tukey’s post hoc test. **D** Inflammatory cell infiltrate was quantified and analyzed by two-way ANOVA with Sidak’s multiple-comparison test

Micro-CT evaluation at day 28 confirmed hard tissue deposition within the pulp chambers of every treatment group (Fig. 1A). The most significant hard tissue formation was recorded in the MTA-only group (0.090 ± 0.021 mm^3^), closely followed by the GelMA/AV + MTA group (0.086 ± 0.029 mm^3^); these values were statistically similar (*p* = 0.961). Animals treated with GelMA/AV alone generated significantly less mineralized tissue (0.023 ± 0.018 mm^3^; *p* < 0.05 vs. both MTA-containing groups; Fig. 1B). Tissue mineral density (TMD) showed the same pattern: MTA (1002 ± 73 g/cm^3^) and GelMA/AV + MTA (1042 ± 37 g/cm^3^) were comparable (*p* = 0.396), whereas GelMA/AV presented a significantly lower TMD (973 ± 26 g/cm^3^; *p* < 0.05 vs. either MTA group; Fig. 1C).

### Histological evaluation of inflammatory response

**Fig. 2:**
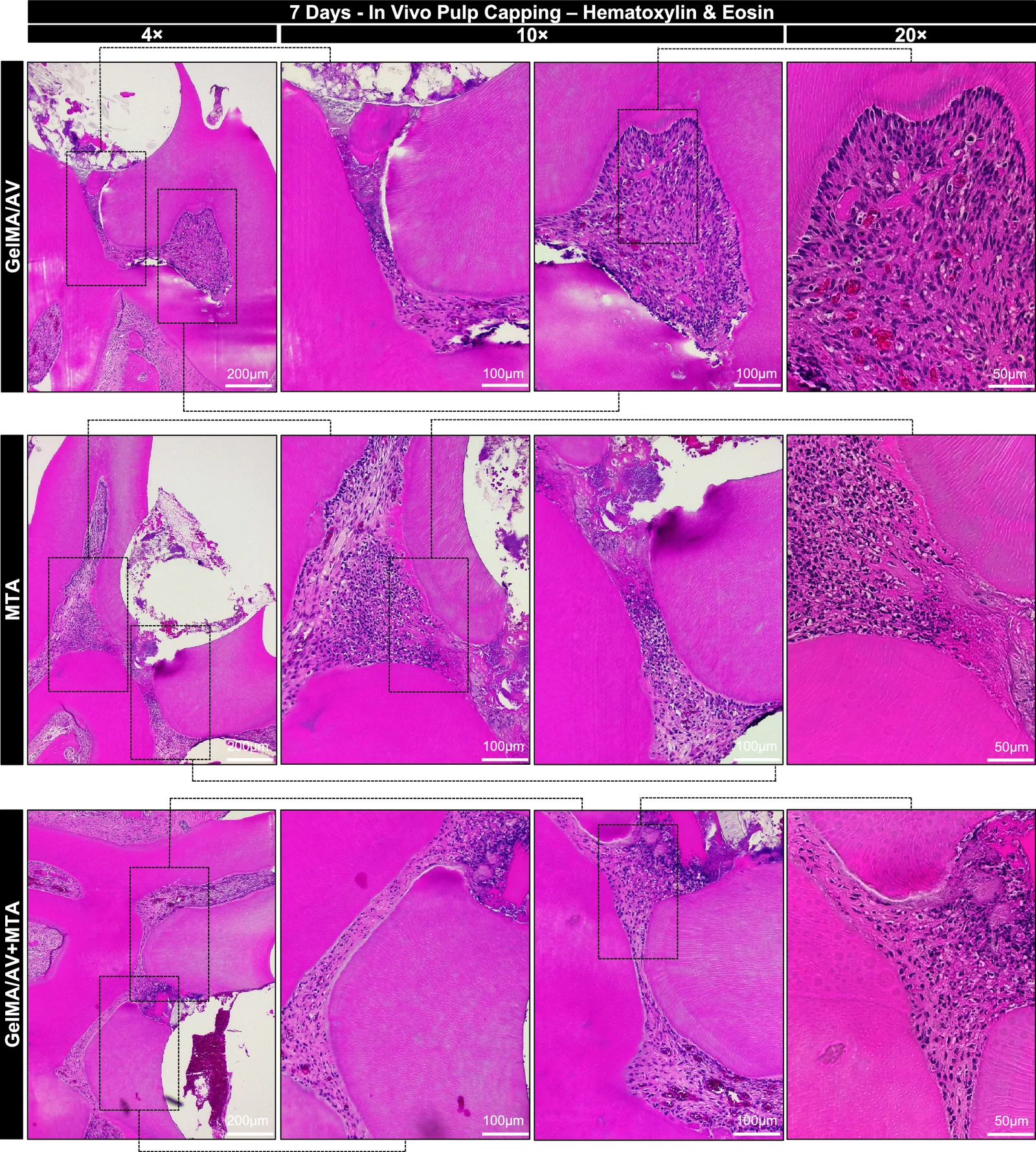
H&E staining 7 days after pulp capping shows the inflammatory cell infiltrate at three magnifications (4 × , 10 × , and 20 ×) for GelMA/AV, MTA, and GelMA/AV + MTA treatments. A similar inflammatory profile and density can be observed in the pulp chamber for all groups. Scale bar: 200 µm for 4 × , 100 µm for 10 × , and 50 µm for 20 ×

**Fig. 3:**
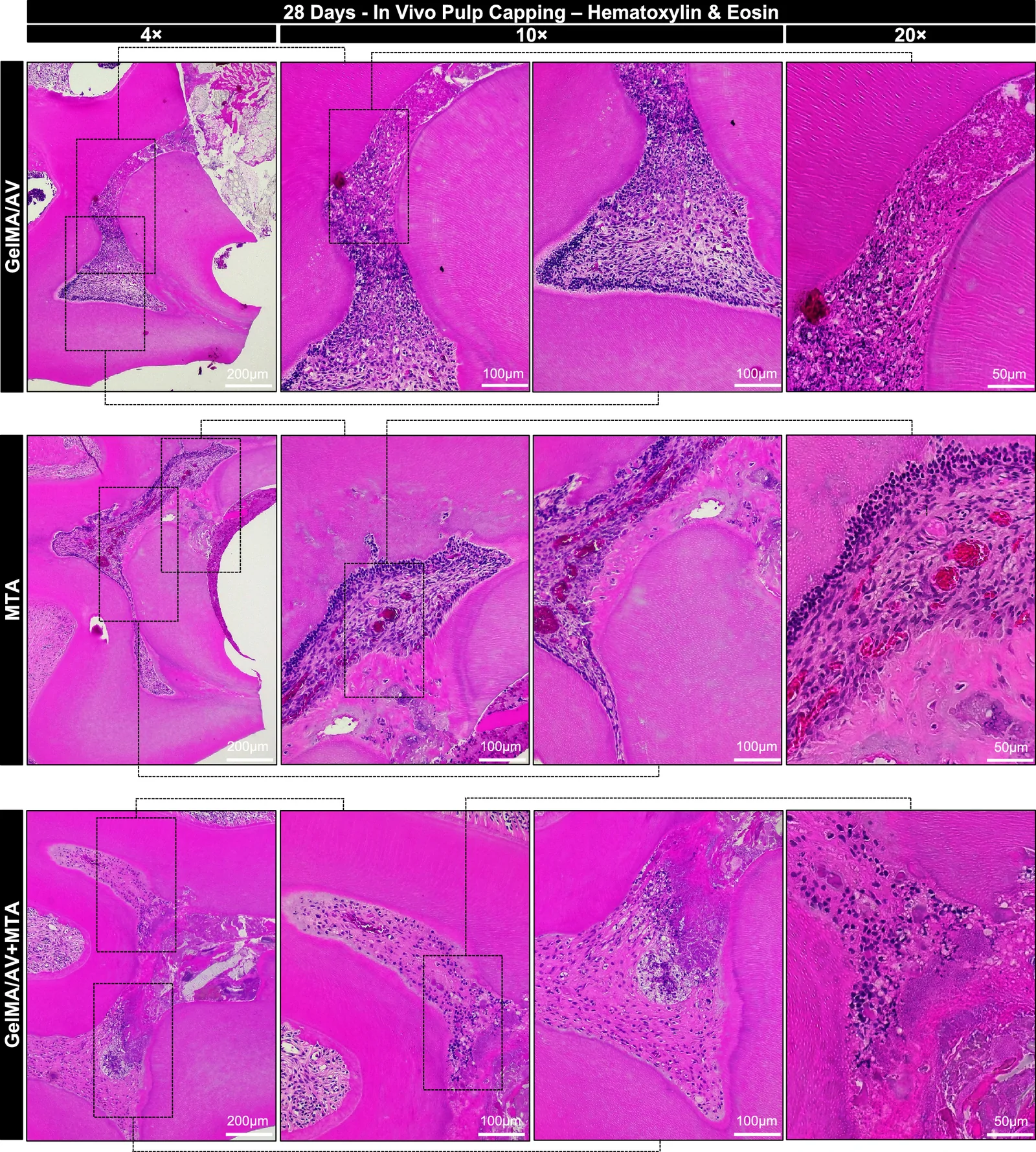
H&E staining 28 days after pulp capping shows the inflammatory cell infiltrate at three magnifications (4 × , 10 × , and 20 ×) for GelMA/AV, MTA, and GelMA/AV + MTA treatments. Observe a diminished cell density when GelMA/AV was applied beneath the MTA layer. Scale bar: 200 µm for 4 × , 100 µm for 10 × , and 50 µm for 20 ×

H&E sections at 7 days revealed a moderate inflammatory infiltrate in every group, ranging from roughly 1600 to 1850 cells/mm^2^, with no significant inter-group differences (*p* > 0.05; Figs. 1D and 2). By 28 days, however, inflammation had subsided most markedly in the GelMA/AV + MTA specimens, which showed only 875 ± 573 cells/mm^2^, significantly fewer than either the MTA group (1721 ± 338 cells mm^2^, *p* = 0.006) or the GelMA/AV group (1921 ± 356 cells mm^2^, *p* = 0.0008; Figs. 1D and 3). The cell density in GelMA/AV-treated animals did not differ from that in MTA alone (*p* = 0.806). On qualitative assessment of the histological findings, vacuolar degeneration was similar across groups and decreased over time, from mild at 7 days to slight at 28 days, likely reflecting inherent features of the animal pulp model. By 28 days, specimens treated with MTA alone exhibited greater pulp hyperemia than those in which GelMA/AV was applied underneath.

### Immunohistochemical analysis of mineralization and macrophage polarization

**Fig. 4:**
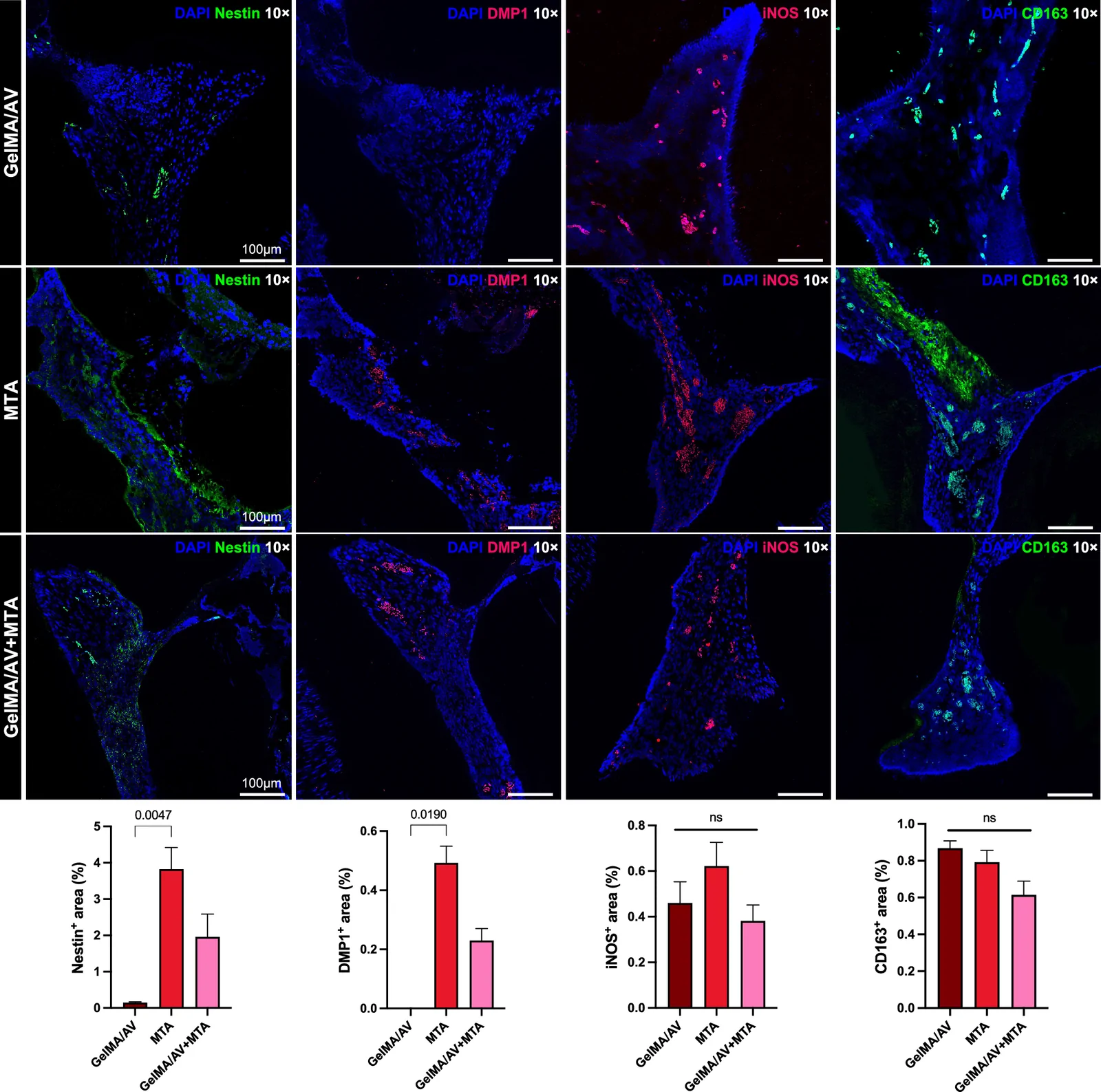
Immunohistochemical evaluation of pulpal tissue 28 days after pulp capping. Representative 10 × images (scale bar, 50 µm) showing staining for Nestin, DMP1, iNOS, and CD163 across the treatment groups. Quantitative analysis of each marker, expressed as the mean ± SD percentage of positively labeled area. Statistical tests: Nestin, iNOS, and CD163: one-way ANOVA with Tukey’s multiple-comparison post-test; DMP1: Kruskal–Wallis test with Dunn’s post-test

By day 28, immunostaining for the odontogenic markers nestin and DMP1 showed the superior mineralizing activity of the MTA-containing treatments. Both the MTA-only and GelMA/AV + MTA groups displayed strong labeling for these proteins, with the MTA group showing approximately twice the positive area of the combined treatment; however, the difference was not statistically significant (nestin, *p* = 0.084; DMP1, *p* = 0.517). In contrast, the GelMA/AV scaffold alone produced only limited nestin signal (0.14 ± 0.03%) and no detectable DMP1, indicating minimal mineralization potential (*p* = 0.0047 and 0.0190 vs. MTA for nestin and DMP1, respectively). Regarding macrophage phenotype, at day 28, immunofluorescence analysis revealed a clear, though statistically non-significant, expression when MTA was applied over the GelMA/AV scaffold. Specifically, the GelMA/AV + MTA group exhibited the smallest iNOS-positive (M1) area, indicating the lowest pro-inflammatory activity. This benefit, however, was accompanied by a concomitant decrease in CD163-positive (M2) staining, suggesting a reduction in the presence of anti-inflammatory macrophages as well (Fig. 4).

## Discussion

In this in vivo study, a photocrosslinkable GelMA nanofiber scaffold loaded with aloe vera (GelMA/AV) as a pulp capping biomaterial, applied either by itself or as a base layer beneath MTA in rat molars with standardized pulp exposures, was examined. The combined strategy, GelMA/AV followed by MTA, noticeably

preserved the robust mineralization classically associated with MTA and reduced overall cellular density at 28 days; however, this metric does not, by itself, equate to reduced inflammation. Consistent with that, macrophage immunophenotyping (iNOS/M1 and CD163/M2) showed directional trends without statistical significance, indicating no demonstrable anti-inflammatory effect of the GelMA/AV sublayer under the conditions tested.

Vital pulp therapy, particularly pulp capping, aims to preserve the tooth’s pulp vitality after exposure by creating conditions for repair and a protective hard tissue barrier to form. The MTA is widely used for this purpose because it strongly promotes hard tissue formation and is highly biocompatible with pulp tissue [19, 20]. However, a key limitation of MTA is that it causes early-stage pulpal inflammation [21]. This limitation provides the rationale for adjunct strategies, such as placing an anti-inflammatory GelMA/AV scaffold beneath the MTA, which pairs the MTA’s reparative strengths with improved inflammatory control to create a more favorable healing environment.

GelMA scaffolds provide an excellent cell-adhesive matrix and are readily degraded by the matrix metalloproteinases that surge after pulp exposure. Yet, in its native form, it neither reduces inflammation nor promotes mineralization, limiting its use as a stand-alone capping agent. To provide a scaffold with antimicrobial and immunomodulatory activity without introducing antibiotics that could foster resistance, a GelMA nanofibers embedded with aloe vera have been developed. In earlier work, this GelMA/AV scaffold suppressed *E. faecalis* biofilms and shifted macrophages toward an M2 profile when implanted in rats’ subcutaneous tissue [9]. In the present in vivo study, placing the GelMA/AV membrane beneath MTA significantly reduced the cellular infiltrate by day 28 compared with either material applied alone. Immunohistochemical data, however, showed that the composite did not alter macrophage polarization toward an M2 phenotype further. Thus, while GelMA/AV appears to reduce cellular density, its capacity to modulate the macrophage phenotype was not evidenced.

Micro-CT assessment revealed that the GelMA/AV scaffold alone produced the least mineralized repair, displaying the lowest hard tissue formation and TMD. In contrast, both MTA-containing groups generated markedly more hard tissue, reaffirming MTA’s status as the benchmark material for direct pulp capping [20, 22, 23]. The hard tissue formation and TMD values for the GelMA/AV + MTA combination were statistically similar to those of MTA alone, showing that the GelMA/AV layer neither enhances nor compromises MTA-driven mineralization. Interestingly, the combination group trended toward slightly lower hard tissue formation but higher TMD than MTA alone; although not significant, this pattern may reflect a slower, more orderly mineral deposition promoted by the scaffold’s anti-inflammatory effect, potentially yielding a denser and better-organized calcific bridge.

Nestin and DMP1 are biomarkers of the pulp’s mineralization response. Specifically, nestin, an intermediate filament protein, is widely recognized as a marker of odontoblast differentiation and reparative dentinogenesis. In healthy mature teeth, nestin is largely confined to fully differentiated, functioning odontoblasts, but it is re-expressed in pulp stem cells when new odontoblast-like cells form in response to injury, such as during tertiary dentin formation after pulp capping. This makes nestin a sensitive indicator that a pulp capping material has stimulated odontoblastic differentiation at the exposure site [24–27]. Moreover, DMP1 is a non-collagenous extracellular matrix protein crucial for dentin mineralization, recognized for its calcium-binding ability and its role in hydroxyapatite deposition within the collagen matrix. It is highly expressed in dentin, indicating mineralizing matrix and regulating odontoblast activity and mineral deposition [28, 29].

Nestin and DMP1 served as markers to evaluate whether the GelMA/AV scaffold enhances MTA’s innate mineralizing capacity. Quantitative analysis revealed that the sequential GelMA/AV + MTA treatment did not elevate either marker above the levels achieved by MTA alone, indicating that the scaffold layer neither enhanced nor inhibited MTA-driven mineralization. Conversely, teeth capped with GelMA/AV without MTA exhibited virtually no DMP1 and only minimal nestin labeling, emphasizing the limited odontogenic potential of the AV-loaded scaffold on its own. These findings contrast with an earlier report in which *acemannan*, an AV-derived compound, was applied directly to exposed pulps and produced a homogeneous calcified bridge [30]. The discrepancy likely reflects differences in formulation and delivery: in the present work, AV was embedded within crosslinked GelMA nanofibers, whereas the prior study applied the soluble extract directly to the tissue, potentially yielding higher local concentrations and faster interaction with dental pulp cells.

This study has important limitations. The rat model, while useful for screening, does not fully recapitulate the microbial complexity or chronicity of human pulpitis. The single 28-day endpoint captures early repair rather than long-term dentin bridge stability [31]. Finally, only one aloe vera concentration and delivery approach was tested; more potent or better-timed immunomodulation may require formulation refinement. Future work should include earlier and longer follow-ups, expanded immunolabeling, and systematic variation of AV dose/chemistry (and/or added bioactives), all within workflows that preserve the simplicity of calcium silicate cement-based VPT. In summary, placing a thin GelMA/AV sublayer beneath MTA preserved MTA’s characteristic mineralization and showed suggestive, but not definitive, signs of an improved inflammatory milieu at 28 days. These findings are hypothesis generating rather than confirmatory and warrant further investigation before any consideration of clinical adoption.

## Data Availability

Data can be provided upon reasonable request.

## References

[1] Duncan HF, Galler KM, et al. European Society of Endodontology position statement: management of deep caries and the exposed pulp. Int Endod J. 2019;52:923–34. 10.1111/iej.13080. 30664240

[2] Witherspoon DE. Vital pulp therapy with new materials: new directions and treatment perspectives–permanent teeth. J Endod. 2008;34:S25–8. 10.1016/j.joen.2008.02.030. 18565368

[3] Parirokh M, Torabinejad M. Mineral trioxide aggregate: a comprehensive literature review–Part I: chemical, physical, and antibacterial properties. J Endod. 2010;36:16–27. 10.1016/j.joen.2009.09.006. 20003930

[4] Alqaderi HE, Al-Mutawa SA, Qudeimat MA. MTA pulpotomy as an alternative to root canal treatment in children’s permanent teeth in a dental public health setting. J Dent. 2014;42:1390–5. 10.1016/j.jdent.2014.06.007. 24973732

[5] Camilleri J. Hydration mechanisms of mineral trioxide aggregate. Int Endod J. 2007;40(6):462–70. 10.1111/j.1365-2591.2007.01248.x. 17459120

[6] Okiji T, Yoshiba K. Reparative dentinogenesis induced by mineral trioxide aggregate: a review from the biological and physicochemical points of view. Int J Dent. 2009;2009:464280. 10.1155/2009/464280. PMC2837314 20339574

[7] Fouad AF. Microbial factors and antimicrobial strategies in dental pulp regeneration. J Endod. 2017;43:S46–50. 10.1016/j.joen.2017.06.010. 28778502

[8] Galler KM, D’Souza RN, Federlin M, Cavender AC, Hartgerink JD, Hecker S, et al. Dentin conditioning codetermines cell fate in regenerative endodontics. J Endod. 2011;37(11):1536–41. 10.1016/j.joen.2011.08.027. 22000458

[9] Namazi SS, Mahmoud AH, Dal-Fabbro R, Han Y, Xu J, Sasaki H, et al. Multifunctional and biodegradable methacrylated gelatin/*Aloe vera* nanofibers for endodontic disinfection and immunomodulation. Biomater Adv. 2023;150:213427. 10.1016/j.bioadv.2023.213427. PMC11027083 37075551

[10] Gao Y, Kuok KI, Jin Y, Wang R. Biomedical applications of *Aloe vera*. Crit Rev Food Sci Nutr. 2019;59:S244–56. 10.1080/10408398.2018.1496320. 29999415

[11] Sanchez M, Gonzalez-Burgos E, Iglesias I, Gomez-Serranillos MP. Pharmacological update properties of *Aloe vera* and its major active constituents. Molecules. 2020. 10.3390/molecules25061324. PMC7144722 32183224

[12] Vijayalakshmi D, Dhandapani R, Jayaveni S, Jithendra PS, Rose C, Mandal AB. In vitro anti inflammatory activity of *Aloe vera* by down regulation of MMP-9 in peripheral blood mononuclear cells. J Ethnopharmacol. 2012;141(1):542–6. 10.1016/j.jep.2012.02.040. 22446321

[13] Budai MM, Varga A, Milesz S, Tozser J, Benko S. *Aloe vera* downregulates LPS-induced inflammatory cytokine production and expression of NLRP3 inflammasome in human macrophages. Mol Immunol. 2013;56:471–9. 10.1016/j.molimm.2013.05.005. 23911403

[14] Yue K, Trujillo-de Santiago G, Alvarez MM, et al. Synthesis, properties, and biomedical applications of gelatin methacryloyl (GelMA) hydrogels. Biomaterials. 2015;73:254–71. 10.1016/j.biomaterials.2015.08.045. PMC4610009 26414409

[15] Han Y, Dal-Fabbro R, Mahmoud AH, et al. GelMA/TCP nanocomposite scaffold for vital pulp therapy. Acta Biomater. 2024;173:495–508. 10.1016/j.actbio.2023.11.005. PMC10964899 37939819

[16] Anselmi C, Mendes Soares IP, Dal-Fabbro R, et al. Multifunctional bilayer scaffold for dental pulp protection and sustained calcium hydroxide release for mineralized tissue regeneration. Biomaterials. 2025;326:123700. 10.1016/j.biomaterials.2025.123700. PMC13033331 40974742

[17] Reis-Prado AHD, Rahimnejad M, Dal-Fabbro R, Toledo PTAd, Anselmi C, Oliveira PHCd, et al. Injectable thermosensitive antibiotic-laden chitosan hydrogel for regenerative endodontics. Bioact Mater. 2025;46:406–22. 10.1016/j.bioactmat.2024.12.026. PMC11754974 39850022

[18] Dal-Fabbro R, Yu M, Mei L, et al. Synthetic high-density lipoprotein (sHDL): a bioinspired nanotherapeutics for managing periapical bone inflammation. Int J Oral Sci. 2024;16:50. 10.1038/s41368-024-00316-w. PMC11219839 38956025

[19] Rao A, Rao A, Shenoy R. Mineral trioxide aggregate--a review. J Clin Pediatr Dent. 2009;34:1–7. 10.17796/jcpd.34.1.n1t0757815067g83. 19953801

[20] Camilleri J. Mineral trioxide aggregate in dentistry: from preparation to application. New York: Springer-Verlag Berlin Heidelberg; 2014.

[21] Reyes-Carmona JF, Santos AS, Figueiredo CP, Baggio CH, Felippe MCS, Felippe WT, et al. Host-mineral trioxide aggregate inflammatory molecular signaling and biomineralization ability. J Endod. 2010;36(8):1347–53. 10.1016/j.joen.2010.04.029. 20647094

[22] Parirokh M, Torabinejad M. Mineral trioxide aggregate: a comprehensive literature review–Part III: clinical applications, drawbacks, and mechanism of action. J Endod. 2010;36:400–13. 10.1016/j.joen.2009.09.009. 20171353

[23] Alsubait S, Aljarbou F. Biodentine or mineral trioxide aggregate as direct pulp capping material in mature permanent teeth with carious exposure? A systematic review and meta-analysis. Oper Dent. 2021;46(6):631–40. 10.2341/20-277-LIT. 35507905

[24] Puspita S, Utoro T, Haniastuti T. Nestin expressions of exposed pulp after direct pulp capping by calcium hydroxide and platelet rich plasma. Eur J Dent. 2016;10:341–4. 10.4103/1305-7456.184157. PMC4926585 27403050

[25] About I, Laurent-Maquin D, Lendahl U, Mitsiadis TA. Nestin expression in embryonic and adult human teeth under normal and pathological conditions. Am J Pathol. 2000;157(1):287–95. 10.1016/S0002-9440(10)64539-7. PMC1850197 10880398

[26] Nakatomi M, Quispe-Salcedo A, Sakaguchi M, et al. Nestin expression is differently regulated between odontoblasts and the subodontoblastic layer in mice. Histochem Cell Biol. 2018;149:383–91. 10.1007/s00418-018-1651-3. 29445893

[27] Kuratate M, Yoshiba K, Shigetani Y, et al. Immunohistochemical analysis of nestin, osteopontin, and proliferating cells in the reparative process of exposed dental pulp capped with mineral trioxide aggregate. J Endod. 2008;34:970–4. 10.1016/j.joen.2008.03.021. 18634929

[28] Yamada M, Nagayama M, Miyamoto Y, et al. Mineral Trioxide Aggregate (MTA) upregulates the expression of DMP1 in direct pulp capping in the rat molar. Materials. 2021. 10.3390/ma14164640. PMC8400143 34443162

[29] Beniash E, Deshpande AS, Fang PA, et al. Possible role of DMP1 in dentin mineralization. J Struct Biol. 2011;174:100–6. 10.1016/j.jsb.2010.11.013. PMC3073716 21081166

[30] Jittapiromsak N, Sahawat D, Banlunara W, Sangvanich P, Thunyakitpisal P. Acemannan, an extracted product from *Aloe vera*, stimulates dental pulp cell proliferation, differentiation, mineralization, and dentin formation. Tissue Eng Part A. 2010;16:1997–2006. 10.1089/ten.TEA.2009.0593. 20088703

[31] Celik BN, Mutluay MS, Arikan V, Sari S. The evaluation of MTA and Biodentine as a pulpotomy materials for carious exposures in primary teeth. Clin Oral Investig. 2019;23:661–6. 10.1007/s00784-018-2472-4. 29744721

